# BUILDING AN ELEPHANT BRAIN DATABASE: ADVANCING INSIGHTS INTO COGNITIVE AGING IN ELEPHANTS

**DOI:** 10.1093/geroni/igae098.3638

**Published:** 2024-12-31

**Authors:** Daniella Chusyd, Patrick Hof, Shannon Risacher, Hu Cheng, Chet Sherwood

**Affiliations:** Indiana University, Bloomington, Indiana, United States; Mount Sinai, New York, New York, United States; Indiana University, Indianapolis, Indiana, United States; Indiana University, Indiana University, Indiana, United States; The George Washington University, Washington DC, District of Columbia, United States

## Abstract

Cognitive aging is a complex phenomenon, and a comparative approach leverages the strengths of various species to identify conserved and divergent mechanisms associated with cognitive health. Elephants are an informative species for cognitive aging research because they are analogous to humans in that they live to comparable ages, display complex cognition, have strong long-term memory, and rely on social bonds. They differ, however, in their evolutionary path to arrive at these same outcomes. Studying elephants can potentially reveal different trajectories for cognitive performance maintenance and impairment compared to humans, and provide information on brain aging in elephants, contributing missing knowledge on aspects of well-being in a long-lived species. We initiated the Elephant Brain Database to enhance the use of elephants as a comparative species to further understand the structure, function, and evolution of the brain, and to increase the availability of elephant postmortem brain tissue, MRI scans, and related datasets for investigators. This repository is for postmortem fixed and frozen brain specimens and when possible, postmortem structural MRI scans and diffusion tensor images. In collaboration with North American zoos and partners in Zambia, we obtained fixed or frozen brain specimens from 23 individuals (age: stillborn to 75 years). Of those, 12 are from Asian elephants (Elephas maximus; 8 females, 4 males) and 11 are from African savanna elephants (Loxodonta africana; 4 females, 7 males). A structural MRI scan was conducted on one left hemisphere from a 52-year-old female Asian elephant. This collection represents the largest consolidation of elephant brain resources.

